# When mirroring is both simple and “smart”: how mimicry can be embodied, adaptive, and non-representational

**DOI:** 10.3389/fnhum.2014.00505

**Published:** 2014-07-14

**Authors:** Evan W. Carr, Piotr Winkielman

**Affiliations:** ^1^Department of Psychology, University of California - San DiegoLa Jolla, CA, USA; ^2^Department of Cognitive Science, University of California - San DiegoLa Jolla, CA, USA; ^3^Faculty of Psychology, University of Social Sciences and HumanitiesWarsaw, Poland

**Keywords:** embodiment, imitation, social learning, mimicry, mirror neurons

## Abstract

The concept of *mirroring* has become rather ubiquitous. One of the most fundamental empirical and theoretical debates within research on mirroring concerns the role of mental representations: while some models argue that higher-order representational mechanisms underpin most cases of mirroring, other models argue that they only moderate a primarily non-representational process. As such, even though research on mirroring—along with its neural substrates, including the putative *mirror neuron system*—has grown tremendously, so too has confusion about what it actually means to “mirror”. Using recent research on spontaneous imitation, we argue that flexible mirroring effects can be fully embodied and dynamic—even in the *absence* of higher-order mental representations. We propose that mirroring can simply reflect an adaptive integration and utilization of cues obtained from the brain, body, and environment, which is especially evident within the social context. Such a view offers reconciliation among both representational and non-representational frameworks in cognitive neuroscience, which will facilitate revised interpretations of modern (and seemingly divergent) findings on *when* and *how* these embodied mirroring responses are employed.

## Introduction

There is now great interest in the idea of an *embodied* mind. Much evidence suggests that thought and action are grounded in perceptual and sensorimotor states, while being shaped by the environment in which they take place (e.g., Niedenthal et al., 2005). But how does this grounding and shaping occur, what are the regulatory mechanisms, and when (if ever) are *higher-order representations* necessary? In the current article, we explore these questions in the context of debates about the role of mirroring in social cognition.

One dominant idea guiding embodiment research on social cognition is that individuals understand others by replicating their states, using one’s own somatosensory resources. On the neural level, this function is often assigned to the putative *mirror neuron system* (MNS), which “re-creates” the observed action in the perceiver (Blakemore and Decety, 2001; Gallese, 2003a; Rizzolatti and Craighero, 2004). Several mirroring accounts appeal to higher-order representational processes (e.g., Goldman and Sripada, 2005), and while definitions of “mental representations” certainly do vary, those models usually view them as outcomes of propositional encoding of incoming sensory information into language-like mental symbols, which can then be internally manipulated, and finally back-translated into motoric processes that lead to action (Gallese, 2003a,b). We will return to these definitional issues later.

Critically, what makes the “mirroring” idea and related phenomena appealing is the possibility that they reveal a non-representational relation to others (Gallagher, 2007; Hutto, 2007; Sinigaglia, 2009). With the following, we illustrate and expand on this general point, using recent literature on *spontaneous imitation* (or the reflexive mimicry of another’s actions or behaviors). Such imitation—of gestures, finger movements, facial expressions, etc.—plays a role in empathy, affiliation, and rapport (Chartrand and van Baaren, 2009). Importantly, this imitation can sometimes be direct (e.g., smile-to-a-smile, index-finger to index-finger, etc.), possibly reflecting operation of low-level ideomotor processes (Brass et al., 2000; Gallese, 2003a; Catmur et al., 2009).

Note, however, that non-representational accounts of mirroring face a basic problem, when imitation is *in*direct: as we will elaborate on shortly, perceivers do indeed faithfully “mirror” or replicate the observed state—but only *sometimes*. In fact, individuals often engage in adaptable, context-sensitive responding, whereby this direct perceiver-observer correspondence goes beyond a mere reflection of what is observed. This flexibility is often highlighted by critics of the non-representational accounts to embodiment, as presumably necessitating mediation by higher-order representations. Indeed, some recent theories of imitation explicitly suggest that such contextual modification reveals the mediating role of higher-order constructs, such as sophisticated appraisals, meaning-construction, and even theory-of-mind (Wang and Hamilton, 2012; Hess and Fischer, 2013).

Consequently, to address the problem of indirect imitation, any sophisticated non-representational account needs to explain how perceivers’ responses to the observed action reflect dynamic integration of perception-action links, bodily states, and social-environmental cues (Chemero, 2009). Such accounts should explain how context-dependent shaping of embodiment can not only occur quickly, implicitly, and with little conscious awareness, but also how this can manifest even in animals (de Waal and Ferrari, 2010; Barrett, 2011) and “embodied” robots (Wilson and Golonka, 2013), which have limited capacities for higher-order mental representations. If successful, this suggests an exciting, novel alternative perspective on the flexibility of mirroring, where higher-order representations are not necessary.

Our view assumes that flexible mirroring effects can be fully embodied, dynamic, and adaptive—even in the absence of higher-order mental representations. We propose that spontaneous mirroring can be “smart”, in the sense that the process can simply and efficiently utilize integrative environmental cues. To demonstrate this, we offer a snapshot of the recent findings on spontaneous imitation, situated within this debate about the mediating role of higher-order mental representations.

## Arguments for representational models

Many representational arguments for mirroring arose during initial interpretational debates regarding neurons in ventral premotor cortex that discharge both during action-performance and action-observation (originally, in area F5 of the rhesus macaque; Rizzolatti and Craighero, 2004). The popular early “adaptationist” frameworks argue that the MNS was evolutionarily selected to subserve action-understanding and promote social learning (Iacoboni, 2009). Importantly, different models within this broad domain vary in their explanation of the underlying mechanisms for how mirroring is instantiated and recruited. For instance, some argue that the observer has a common cognitive representational format for perception and action (i.e., active intermodal matching models; Preston and De Waal, 2002; Meltzoff and Decety, 2003), that mirroring is dependent on internal re-creations of others’ states in order to facilitate understanding and co-regulation (i.e., simulationist and embodiment frameworks; Goldman and Sripada, 2005; Semin and Cacioppo, 2009), or that imitation is fundamentally shaped by higher-level meaning-constructions (i.e., social and emotional frameworks; Hamilton, 2013; Hess and Fischer, 2013; Reed and McIntosh, 2013). Critically though, while these models differ in how they explain the function of the MNS (and the resultant tendencies toward mirroring), all agree that the cognitive and neural resources that allow such mirroring to occur are substantially dependent on higher-order representations, which then act to advance action-learning, recognition, and understanding.

**Figure 1:**
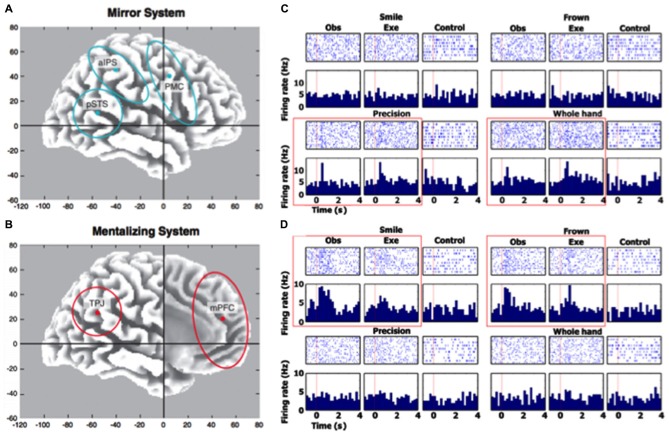
**Human neural responses when mirroring and responding to others.** Panels **A** and **B** (figure and caption are reprinted from Van Overwalle and Baetens (2009) with permission from Elsevier) depict the regions of interest involved in the mirror and mentalizing system placed in an x–y–z Talairach atlas. Their centers are indicated by a dot and include pSTS (±50 −55 10), TPJ (±50 −55 25), aIPS (±40 −40 45), PMC (±40 5 40) and mPFC (0 50 20). The PC (with center 0 −60 40) is not shown. The regions are drawn based on the recent literature and, in particular, on Keysers and Gazzola (2006; panel **A**) for the mirror system and on Van Overwalle (2009; panel **B**) for the mentalizing system. Panels **C** and **D** (figure and caption are reprinted from Mukamel et al. (2010) with permission from Elsevier) show neural responses of two cells during all experimental conditions and tasks, which depicts an action observation/execution matching multiunit in left SMA for the two grips (precision and whole-hand; panel **C**) and an action observation/execution matching single unit in right entorhinal cortex for two facial gestures (smile and frown; panel **D**). Rasters (top) are aligned to stimulus onset (red vertical line at time = 0). Bin size for peristimulus time histogram (bottom) is 200 ms. Red box highlights responses passing statistical criteria.

Arguably some of the strongest pro-representational arguments came from the interpretation of studies showing that our imitative capacities are “smart”—that is, mirroring responses dynamically adapt to environmental cues, for both motor and facial behaviors. As examples, motor imitation (e.g., finger-lifting, hand-opening and closing, etc.) is modulated by prosocial attitudes (Leighton et al., 2010b), incidental similarity (Guéguen and Martin, 2009), affiliative drive (Lakin and Chartrand, 2003), and even eye contact (Wang et al., 2011). Interestingly, emotional imitation exhibits an even greater bit of flexibility: for example, spontaneous facial mimicry of emotional expressions (i.e., smile-to-a-smile, frown-to-a-frown, etc.) can change dramatically according to social factors, like outgroup membership (Bourgeois and Hess, 2008), negative attitudes (Likowski et al., 2008), competition (Weyers et al., 2009), and social power (Carr et al., 2014). Since many of these studies use sensitive psychophysiological techniques (e.g., electromyography [EMG] to gauge motor activity over different facial muscles; e.g., Carr et al., 2014) and neuroscientific methods (e.g., single-unit recordings in humans; Mukamel et al., 2010; see Figures 1C,D), these findings suggest that even at the lowest level, facial mimicry is sensitive to contextual cues, basic appraisals, and rudimentary goal processes (Hess and Fischer, 2013). Crucially, the representational models posit that higher-order constructs are required for such contextual modifications to occur.

Modern research in neuroscience has furthered this representational stance, highlighting the social adaptability of these mirroring responses. Numerous fMRI studies have demonstrated that when we observe another individual’s goal-directed actions (versus other actions with no meaningful intent), traditional ROIs associated with the MNS are activated (e.g., dACC, inferior frontal gyrus (IFG), inferior parietal lobule (IPL), pre-supplementary motor area (preSMA); de Lange et al., 2008; Guionnet et al., 2012), which often co-occur with “mentalizing” ROIs that activate when relating to others (e.g., mPFC, posterior cingulate cortex (PCC), temporoparietal junction (TPJ), Van Overwalle and Baetens, 2009; Becchio et al., 2012; see Figures 1A,B). Similar results have also been observed in interactive dyad studies using EEG, where the social relation to the target modulates key action-related ERP components (e.g., contingent negative variation [CNV]; Kourtis et al., 2010) and μ-wave suppression (Hogeveen et al., 2014; but also see Braadbaart et al., 2013). While none of these studies prove the existence of a mediating role for higher-order representations, they do suggest that we have dedicated cognitive capacities that are tuned to the social relevance of others’ actions.

One interesting aspect of mirroring in the social environment is the possible role of the self (Mitchell, 2009). Specifically, psychological studies show that the self-concept is used as a starting point for attribution of mental states and attitudes to others, supporting the *anchoring-and-adjustment view* (Epley and Gilovich, 2001; Epley et al., 2004; Jenkins et al., 2008; Tamir and Mitchell, 2010). Neurally, mirroring often activates regions (like anterior intraparietal sulcus (aIPS), posterior superior temporal sulcus (pSTS), and PMC; see Figure 1A) that complement the “mentalizing” system (involving the TPJ and MPFC; see Figure 1B). Moreover, studies that use single-pulse TMS (which measure motor-evoked potentials [MEPs]) suggest that social perspective-taking and goal-directedness impacts motor preparation during very early processing stages (Obhi et al., 2011; Sartori et al., 2011; Hogeveen and Obhi, 2012; Cardellicchio et al., 2013; Senna et al., 2014). In sum, it appears that one way mirroring adapts to the social context is via mechanisms that involve the self and that progress in the debate about the representational nature of mirroring could be made by analyzing these mechanisms more in-depth.

Overall, while the above studies do not necessarily show that the mirroring mechanism is inherently representational, they do again suggest that the social nature of observed actions results in the dynamic adjustment of our own “smart” imitation. Partly as a result of such evidence, the non-representational mirroring theories have to be formulated (or reformulated) to explain how context-sensitivity could result less from higher-order representations, and more from simpler variables such as sensorimotor training, perception-action links, and developmental interactions with the social environment—which we move on to next.

## Support for non-representational frameworks

Recently, newer theories have been proposed for mirroring that reverse the logic underlying representational models. For example, one major non-representational framework proposes that “mirror neurons” are formed as a result of a domain-general learning mechanism that pairs contingent and contiguous perception and action (Heyes, 2011). According to this theory, spontaneous imitation can still adapt to context because different perception-action links can be selectively triggered in a particular setting (i.e., contextual modification). Critically however, the MNS does not code the goal-directed nature of observed actions, nor does it necessarily even strictly “mirror” those perceived behaviors (Cook and Bird, 2013; Cook et al., 2014). Other non-representational accounts for mirroring echo this same general sentiment, but instead argue that action-understanding is purely *motoric* in nature and based within the specific neural function of the MNS (Sinigaglia, 2009), which is then developed through supportive environmental interactions like narrative and social observation (Hutto, 2007). Generally though, non-representational frameworks question one of the fundamental aspects of representational models—the involvement of higher-order constructs that map another’s actions onto one’s own body, in order to solve this so-called “correspondence problem” (Iacoboni, 2009).

Much work in psychology and neuroscience on spontaneous imitation in humans appears to support this non-representational perspective. Overall, such studies have shown that mirroring can be quite automatic, yet is quickly reversible and contextually modifiable by low-level perceptual and motor factors. For example, normal patterns of automatic motor imitation can be reversed through brief periods of “counter-mirror training” (Catmur et al., 2007), and simple visual feedback improves facial imitative training (Cook et al., 2013). Furthermore, spontaneous imitation is influenced by low-level stimulus-related features, like visual context and stimulus velocity (Bisio et al., 2010), kinematic fidelity (Eaves et al., 2012), and testing environment (Cook et al., 2012b). Recall that under representational models, mirroring should be goal-directed; however, recent experiments have shown that this process does not always encode behavioral goals (Bird et al., 2007; Leighton et al., 2010a), persisting in social situations even against strong competitive incentives *not* to imitate (Belot et al., 2013) and even in settings when participants are clearly convinced that a model, such as an android, lacks any intentionality (Hofree et al., in press). While this is not necessarily direct evidence for non-representational accounts (since some representations might be unconscious), it definitely argues against the explicit representational notion of goals and intentions—especially given that these mirroring modifications often manifest quickly, unconsciously, and physiologically.

**Figure 2:**
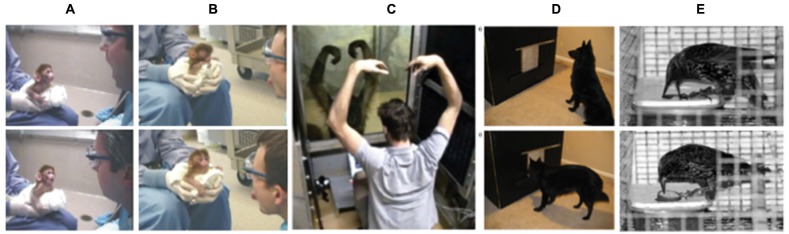
**Demonstrations of spontaneous imitation in non-humans.** Two examples of a neonatal macaque’s imitative response to **(A)** mouth opening and **(B)** tongue protrusion (reprinted from Ferrari et al., 2006). **(C)** Experimenter and female orangutan (*Pongo pygmaeus*) interacting in the contingent/matching condition showing an example of testing behavior (reprinted from Haun and Call (2008) with permission from Elsevier). **(D)** Apparatus with dog at the start of a test trial (top panel), and apparatus as dog is starting to make a screen-push response as test for imitation (bottom panel) (reprinted from Miller et al. (2009) with permission from Elsevier). **(E)** Starlings engaging in a two-action method task for imitative learning, with a push demonstrator (top panel) and a pull demonstrator (bottom panel) (reprinted from Fawcett et al. (2002) with permission from Elsevier).

Further, research on spontaneous imitation in animals provides some of the most convincing evidence for non-representational frameworks, since higher-level representations should presumably be less relevant (or non-existent). In short, humans are not the only ones that can flexibly imitate (see Figure 2): past work has demonstrated spontaneous imitation in primates (Byrne and Tanner, 2006; Ferrari et al., 2006; Bard, 2007; Voelkl and Huber, 2007; Haun and Call, 2008; Dindo et al., 2010), birds (Fawcett et al., 2002; Zentall, 2004; Mui et al., 2008), rats (Heyes et al., 1994), and even dogs (Miller et al., 2009). In fact, even in animals, spontaneous imitation seems to be socially specific and selective. As examples, capuchin monkeys display greater affiliation towards humans who imitate them (Paukner et al., 2009) and dogs can selectively imitate a human behavior that is more preferential in nature (Range et al., 2007). In short, these studies showing contextual imitation in animals challenge the idea that higher-order, explicit representations drive the flexible and efficient social adjustment of imitative responses.

Moreover, other non-human examples of imitation further highlight this possibility of a more “radical”, non-representational mirroring account. For instance, dolphins have been shown to have an advanced capacity for social spontaneous imitation, but this appears to be largely dependent on perception-action interactions with the environment. Specifically, when dolphins are blindfolded, they still copy target behaviors, but they change to a strategy that is more dependent on echolocation (i.e., detecting relevant behaviors through sound), rather than traditional visual routes of perception. Consequently, the authors posit that “such flexibility in changing perceptual routes demonstrates that the dolphin’s imitation was not automatically elicited, but rather results from an intentional, problem-solving approach to imitation [that utilizes the surrounding environment]” (Jaakkola et al., 2013). While numerous examples exist for this perception-action strategy in adjusting to the environment, not all based in the literature on spontaneous imitation (e.g., mate selection by crickets, pack hunting by wolves, etc.; Wilson and Golonka, 2013), the key point here is that social adaptation (including imitative behaviors) can be perceptually grounded without the need for representational mediation, at least in these non-human subjects.

Similarly, other work on “embodied” robots supports this non-representational notion that imitative behaviors can be entrained within the environment (e.g., via hierarchical optimization learning strategies; Billard et al., 2004; Argall et al., 2009). Interestingly, these methods can actually result in socially and emotionally responsive humanoids (Breazeal, 2003). Such spontaneous imitation in robots can even be “goal-directed”, where task behaviors are preferentially selected (Calinon et al., 2005), and these “representations” are developed purely through autonomous interactions with the physical environment (Ijspeert et al., 2002; Wischmann et al., 2006; Gigliotta and Nolfi, 2008). These findings are especially intriguing, given that non-human agents obviously cannot depend on internal cognitive resources, which would be necessary for imitation to occur according to representational models.

In sum, non-representational frameworks emphasize that mirroring responses usually seen in spontaneous imitation are perceptually and motorically grounded within the greater context of the social environment—and *not* solely through the manipulation of higher-order representations. This idea is supported by research demonstrating that spontaneous imitation can occur quickly and implicitly, responding to biologically implausible or impossible actions (Liepelt and Brass, 2010) or persisting in situations that make little sense from a social perspective (Cook et al., 2012a; Hofree et al., in press). Intriguingly, this non-representational mirroring account also follows across other areas of literature, given “smart” imitation findings in non-human agents, like robots and animals (see the following for a review: Huber et al., 2009; de Waal and Ferrari, 2010; Gawronski and Cesario, 2013).

## Conclusion and future directions

In this mini-review, we have addressed a fundamental debate surrounding research and theory on mirroring—the role of mental representations. We argued that spontaneous imitation highlights the flexible, dynamic, yet still embodied aspects of mirroring—even in the absence of higher-order representations. Note that these frameworks do not have to be mutually exclusive on all fronts. For instance, both accounts posit that developmental experience and social context can modify mirroring responses (albeit, to different extents). Thus, while acknowledging that these models contain certain areas of overlap, the most valuable empirical advances will be made in investigating *when* and *how* these divergent imitative patterns manifest.

While this is indeed a difficult question to address, we believe that investigations of mirroring *within the social context* will be most informative: as examples, while non-representational studies have shown modulations of “automatic” imitation through the manipulation of low-level factors (e.g., stimulus-related perceptual cues), such paradigms often strip these stimuli of their real-world social relevance, where mirroring (and the use of representations) could be the most crucial. Also, higher-order representations may be especially useful with mirroring in novel social situations (where “mentalizing” is required), and the self is used to “bootstrap” mental states onto the other person (e.g., Mitchell, 2009; Tamir and Mitchell, 2010). On the other hand, more straightforward social scenarios may not require the instantiation of the self-concept as a reference in mirroring (or its related mental representations). Such an interpretation might suggest that the existence of representations in mirroring responses should not be the primary question, compared to examining how mirroring *transitions* between simple and “smart” states, especially within varying social situations.

Explorations into such questions will prove especially beneficial with newer advances in both technology (e.g., single-unit recordings in humans during action-observation and performance; Mukamel et al., 2010; see Figures 1C,D) and methodology (e.g., using “counter-mirror training” to study imitation in *social* situations; Catmur et al., 2007). Additionally, non-representational frameworks do not have to be “all-or-nothing”, since there are multiple levels of interpretation that can be employed. For instance, while some non-representational mirroring accounts would posit a more “radical” mindset (whereby these responses are purely driven by perception-action interactions with the environment, thus replacing complex internal representations; Wilson and Golonka, 2013), others may argue that higher-order representations are just another moderator of an otherwise non-representational mirroring process. In turn, evaluating the *functional* role of representations in mirroring would be especially informative, where future studies not only explore the co-occurrence of goal-directed “mentalizing” ROIs (e.g., via fMRI), but also what happens to mirroring when these ROIs are *impeded* (e.g., via TMS)?

Going forward, we argue for a flexible embodiment framework that is both simple and “smart”—a view that offers a reconciliation among “traditional” symbolic perspectives in cognitive neuroscience and more “radical”, non-representational theories of embodied processing. Critically, such a mindset will encourage new insights into the interpretation of modern (and seemingly divergent) findings from numerous fields across psychology and neuroscience—particularly on *when* and *how* these embodied imitative responses are engaged.

## Conflict of interest statement

The authors declare that the research was conducted in the absence of any commercial or financial relationships that could be construed as a potential conflict of interest.
